# A hybrid system with highly enhanced graphene SERS for rapid and tag-free tumor cells detection

**DOI:** 10.1038/srep25134

**Published:** 2016-04-27

**Authors:** Ningbo Yi, Chen Zhang, Qinghai Song, Shumin Xiao

**Affiliations:** 1 Integrated Nanoscience Lab, Department of Material Science and Engineering, Shenzhen Graduate School, Harbin Institute of Technology, Shenzhen, 518055, China; 2 Integrated Nanoscience Lab, Department of Electronic and Information Engineering, Shenzhen Graduate School, Harbin Institute of Technology, Shenzhen, 518055, China

## Abstract

By dint of unique physical/chemical properties and bio-compatibility, graphene can work as a building block for a SERS substrate and open up a unique platform for tumor cells detection with high sensitivity. Herein we demonstrate a facile system with highly enhanced surface enhanced Raman spectroscopy of graphene (G-SERS). The system consists of a reduced graphene oxide (rGO) sandwiched by silver and gold nanostructures. Due to the ultrasmall thickness of rGO, the inter-coupling between Ag and Au nanoparticles is precisely controlled and the local field enhancement has been improved to more than 70 times. Associated with the unique chemical mechanism of rGO, the hybrid system has been utilized to identify tumor cells without using any biomarkers. We believe this research will be important for the applications of rGO in cancer screening.

Raman spectroscopy is a reliable technique to understand molecules information with the inelastic scattering process in Raman scattering. In practical applications, the Raman signals are relatively weak due to the small scattering cross-section of molecules. Surface-enhanced Raman scattering (SERS) can enhance the fingerprints of molecules and overcome the limitation of sensitivity in conventional Raman spectroscopy, especially in use of noble metal materials^1^. In SERS researches, electromagnetic mechanism (EM) and chemical mechanism (CM) are well accepted mechanisms. Typically, the enhancement factor from EM is roughly proportional to |*E*|^4^. Strong EM enhancement is usually obtained with electromagnetic “hot spot” with a strongly enhanced local field^2^. The enhancement factor from EM has been improved to be more than 10^8^. To further improve the enhancement factor, the coupling between localized surface plasmons (LSPs) has been considered^1^,^3^,^4^,^5^,^6^. However, the coupling of LSPs is critically sensitive to the separation distance between the LSPs. It has been found to increase dramatically with the decrease of separation distance^7^,^8^. Thus precisely control the separation distance to sub-nanometer scale is a key to further improve the enhancement factor of SERS. Compared with other materials, the thickness of monolayer graphene is well fixed at sub-nanometer level and can be very uniform over a large area. Therefore, combining rGO with the coupling of LSPs can be potentially used to further improve the sensitivity of SERS.

CM is based on the charge transfer and the mixing of molecular orbitals with electronic states between molecules and substrate^9^. In addition to the improved coupling of LSPs, graphene has also been used to enhance SERS via CM due to the high affinity of probe molecules to graphene layer. Because of its smooth surface and atomic thickness with excellent optical transmission, graphene has been well accepted as a novel material for SERS substrate^10^,^11^,^12^, and has been widely applied in molecular selectivity^13^,^14^, dye molecule detection, labeled tumor cell detection^3^,^4^,^5^,^15^, biosensing^16^ *et al.* Among the graphene family, reduced graphene oxide (rGO) is more attractive in bio-compatibility due to its imperfect hexagonal lattice of carbon atoms and sufficient active oxygen sites, which can notably enhance the binding between graphene and target molecule^17^,^18^. The enhancement factor of CM is usually as high as at the order of tens. Therefore, applying rGO in LSPs as G-SERS can not only increase the EM but also bring nice bio-compatibility for CM^19^. Therefore, the improved SERS signals can provide a potential way to rapidly detect and identify tag-free tumor cells in low concentration.

In this research, we numerically propose and experimentally fabricate a hybrid system by sandwiching a monolayer rGO between metallic nanostructures. The monolayer rGO can precisely control the separation between Ag and Au nanostructures, and dramatically enhance the intensities of hot spots via strong intercoupling of localized surface plasmons in both Ag and Au. As a result, the sensitivity of rGO Raman scattering has been promoted over 70 times. Meanwhile, taking the advantage of CM enhancement from the rGO layer, the sensitivity of this hybrid system can be further improved and thus can be utilized to identify tumor cell from normal cell.

## Results

### Structural information

**Figure 1:**
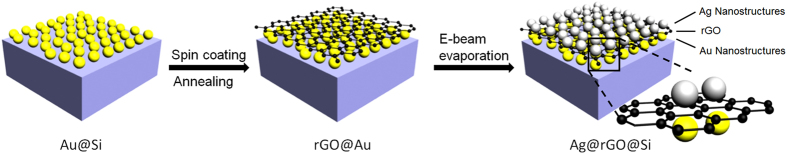
Schematic of the fabrication process for the hybrid system as a G-SERS substrate.

The structure sandwiching rGO between Ag and Au nanostructures (Ag@rGO@Au) is designed and the schematic is shown in Fig. 1 (see the detail fabrication process in Supporting Information). The rGO is obtained using a modified Hummers’ method based on chemistry exfoliated technique^20^,^21^. The thickness of the single GO layer is ~1 nm as demonstrated by the atomic force microscopy (AFM) image in Fig. S1 22. The Au nanostructure layers are deposited on the silicon slide (Au@Si) by electron-beam evaporator with series of thicknesses and the deposition rate of 0.5 A s^−1^. Then the GO solution with concentration of ~6 mg mL^−1^ is spin-coated on the as-prepared substrate of Au@Si, followed by high-temperature annealing in argon gas to obtain rGO (named as rGO@Au). The Ag nanostructure layer is finally deposited on the substrate of rGO@Au using electron-beam evaporator with a detective thickness of 3 nm. The obtained sample is named as Ag@rGO@Au in this research.

### Graphene SERS in plasmonic coupling system

**Figure 2:**
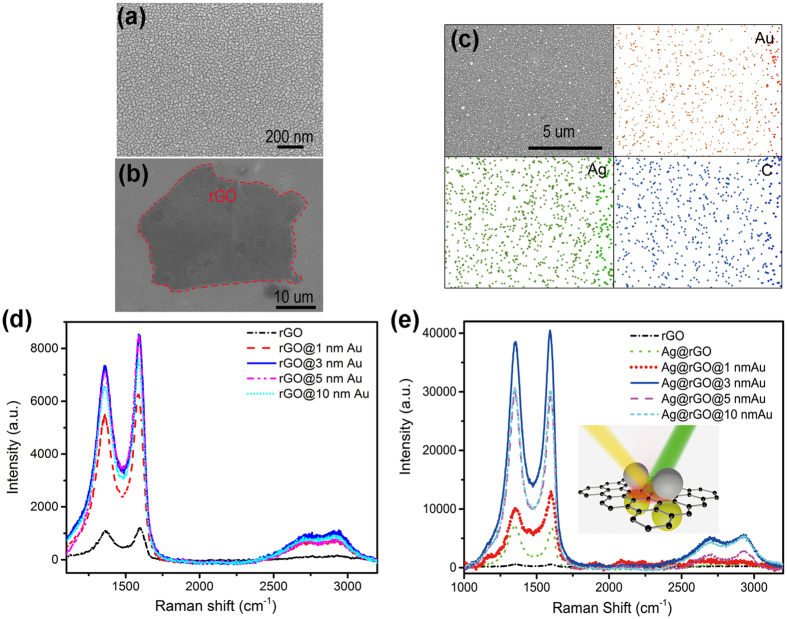
(**a**) SEM image of Au nanostructures with detective thickness of 3 nm (Scale bar: 500 nm). (**b**) SEM image of the substrate of Ag@rGO@Au (Scale bar: 500 nm). The dark region within red line is the area with rGO nanospacer. Enhanced Raman spectra of rGO by different detective thicknesses of Au nanostructures. (**c**) The EDX imaging of Ag, Au and rGO (Red: gold. Green: silver. Blue: carbon). (**d**) Enhanced Raman spectra of rGO by different detective thicknesses of Au nanostructures. Inset: schematic of mechanism of G-SERS. (**e**) Enhanced Raman spectra of rGO by different detective thicknesses of Au nanostructures and 3 nm Ag nanostructures. Inset: schematic of mechanism of G-SERS.

The SEM image of the deposited Au nanostructures on Si substrate is shown in Fig. 2a, where nanocluster morphology on the scale of tens of nanometer can be clearly seen. The Au nanoclusters can excite LSPs and create electromagnetic hot-spots on the surface, which will effectively promote the interaction between light and rGO, to enhance the Raman scattering. The Raman spectra were collected using excitation wavelength of 514.5 nm, and the signal enhancement factor is calculated by comparing the Raman intensity of rGO on the Au nanostructure with that on the blank Si substrate. In Fig. 2d, the two most intensive features associated with rGO, the D peak (~1350 cm^−1^) and G peak (~1580 cm^−1^) are observed, with other two relatively weak peaks corresponding to 2D and 2 G band (~2700 and 2980 cm^−1^ respectively) in accordance with other works^23^,^24^. The Raman peak intensity of rGO@Au can reach over ~7 times higher than that of rGO directly on Si slide. This enhancement is higher than that of graphene@Ag NPs^2^. To achieve the optimal Raman enhancement of rGO, rGO@Au samples are prepared with various Au thicknesses and the corresponding SEM images are shown in Fig. S2. The images show the same nanoclusters morphology with different metal coverage and domain sizes depending on the detective thickness of Au. The UV-vis spectra of Au nanostructure films with different thicknesses are presented in Fig. S3. It can be seen that the reflection value at long wavelength range increases with the increase of Au thickness, indicating more and more metal component exist. Particularly, the Raman signals first rise as the thickness of Au increases and reach maximum at thickness of 3 nm, after which an obvious decline of Raman signals is observed by further increasing the thickness of Au film. The changing trend of Raman signals is consistent with previous studies, indicating that 3 nm is the percolation threshold with existence of largest interconnected nanoclusters and very few individual grains. Near the percolation threshold, the largest average near-field enhancement (hot-spot) can be obtained. Similar study is also performed for Ag@rGO sample with rGO covered by Ag nanostructure layers with different thicknesses. The highest enhancement factor defined as *I*’/*I*_0_ is obtained for Ag layer with thickness of 3 nm with a value of 10 as shown in green dashed line in Fig. 2e, with *I*’ and *I*_0_ denoted as the Raman signal intensity with and without Ag film on top of rGO, respectively^2^.

We further extend our research for a sandwich structure of Ag@rGO@Au to realize coupling between two localized fields to achieve even higher hot spot for Raman enhancement. Ag nanostructures are evaporated on top of the rGO@Au with a detective thickness of 3 nm (Fig. 2b) to obtain sandwiched structures of Ag@rGO@Au. The uniform distribution of Ag, Au and rGO are indicated by performed EDX imaging shown in Fig. 2c. The highly dispersive and uniform of the elements distribution indicates formation of the Ag@rGO@Au sandwiched structure. As shown in Fig. 2e, the D and G peaks of Ag@rGO@Au show greater enhancement than those in rGO@Au. Moreover, the trend of the Raman enhancement is also in agreement with that of rGO@Au, with 3 nm critical thickness for Au nanostructures to obtain maximum Raman signals. The reflection spectra of Ag@rGO@Au samples with different Au thicknesses are plotted in Fig. S4. There are strong reflection peaks covering from 475 nm to 600 nm compared to the spectra for Au@Si in Fig. S3 and Ag@rGO@Si. The reflection peaks result from the coupling of LSPs from Au and Ag nanostructures. Consequently, the coupling system of two LSPS will further enhance the interaction of light with rGO resulting in promoted G-SERS and the enhancement factor is as large as 70.

## Discussion

### Mechanism of G-SERS

**Figure 3:**
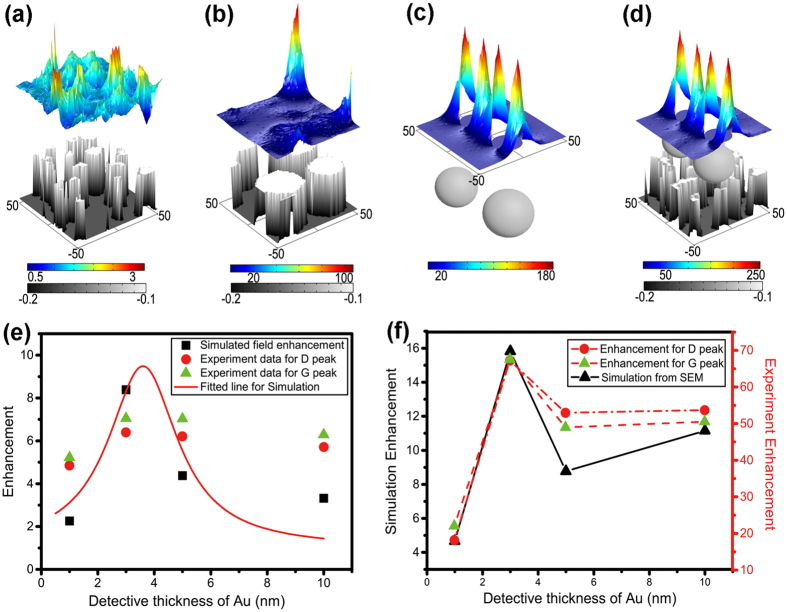
Simulated near-field enhancement and distribution for deep insight of mechanism of G-SERS. The field enhancement is scaled in colors of rainbow and morphology of metal nanostructures is indicated by gray scale. The unit of axis is nm. (**a**) The simulated near-field distribution of Au nanostructures imported from SEM image with detective thickness of 3 nm. (**b**) The simulated near-field distribution of Ag nanostructures imported from SEM image with detective thickness of 3 nm. (**c**) The simulated near-field distribution of two Ag nanoparticles model for simplification of nanostructures in (**b**), with radius of 20 nm and gap of 10 nm. (**d**) The simulated near-field distribution of modeled Ag nanoparticles coupling with Au nanostructures imported from SEM image with detective thickness of 3 nm. (**e**) The experimentally measured D-peak (red dots) and G-peak (green triangle) of enhancement of G-SERS on the substrate of rGO@Au and the simulated enhancement of electromagnetic field of Au nanostructure by importing the SEM image (black square). The solid line is the fitted curve of the simulation results. (**f**) The measured enhancements of D-peak (red dots) and G-peak (green triangle) on the substrate of Ag@rGO@Au and simulated near-field (black square) of hybrid structures of modeled Ag nanoparticles (radius: 20 nm, gap: 10 nm) on Au nanostructures in (**e**).

Although there are many factors to show important effect on G-SERS, such as defects of graphene, environments, and absorbed gas molecules based on CM, EM is main factor to improve G-SERS in our hybrid system. Therefore, for deep insight into the EM effect on the G-SERS, we use a physical tool, COMSOL Multiphysics (see details in Materials and Methods in Supporting Information), to numerically investigate the corresponding enhancements of electric fields by measuring the average vale of |*E*/*E*_0_|^4^ (proportional to measured Raman signals) exactly on the rGO surface, where *E* and *E*_0_ are the averaged electric field with structure and incident light, respectively. The simulated structures are directly imported from the recorded SEM images of Ag and Au nanostructures with thickness of 3 nm with area of 100 × 100 nm^2^. The optical properties of Ag and Au are described by Drude model as previously reported^25^,^26^. As shown in Fig. 3a, the numerical calculation shows that Au nanostructures can efficiently convert the incident light into localized surface plasmon and form hot spots on the surface. The calculated enhancement factors are shown as black squares in Fig. 3e. While the exact values are slightly different from measured values, their trends are very similar and a critical enhancement exists at the thickness of 3 nm. The Ag nanostructures with much larger grain size of nanocluster show similar local field enhancement effect as Au in Fig. 3b, with a much higher enhancement factor than Au nanostructures.

To understand the influence of the interaction between hot spots in Au and Ag layers, we build a model of two Ag particles (gap of 10 nm, radius of 20 nm by simplifying the nanostructures from SEM image in Fig. 2b) and study the corresponding effects on local field enhancement (|*E*/*E*_0_|^4^), as shown in Fig. 3c,d. The size and gap of the Ag bi-nanoparticles is optimized to obtain comparable enhancement factor to that for Ag nanostructure directly imported from SEM image. The average intensity of near-field can also be effectively enhanced to tens of times by the Ag bi-nanoparticles (Fig. 3c). Then the bi-nanoparticles are addressed close to the Au film and the gap distance between Ag nanoparticles and Au layers is set at 1 nm corresponding to thickness of monolayer rGO measured by AFM. Compared with the enhancements with bi-nanoparticles and Au nanostructures, the near-field has been dramatically enhanced and hot spots have been formed in Fig. 3d. The detailed enhancement factor has been plotted as black dots in Fig. 3f. It can be seen that the coupling of surface plasmons of Ag and Au nanostructures can further improve the intensity of hot spots by more than an order of magnitude in agreement with the multiple of enhancements by every single surface plasmon. Besides, the trend of enhancement factor indicates that the localized field enhancement can be optimized by adjusting the gap between particles, while improved EM effects can be obtained by optimization of the detective thickness.

**Figure 4:**
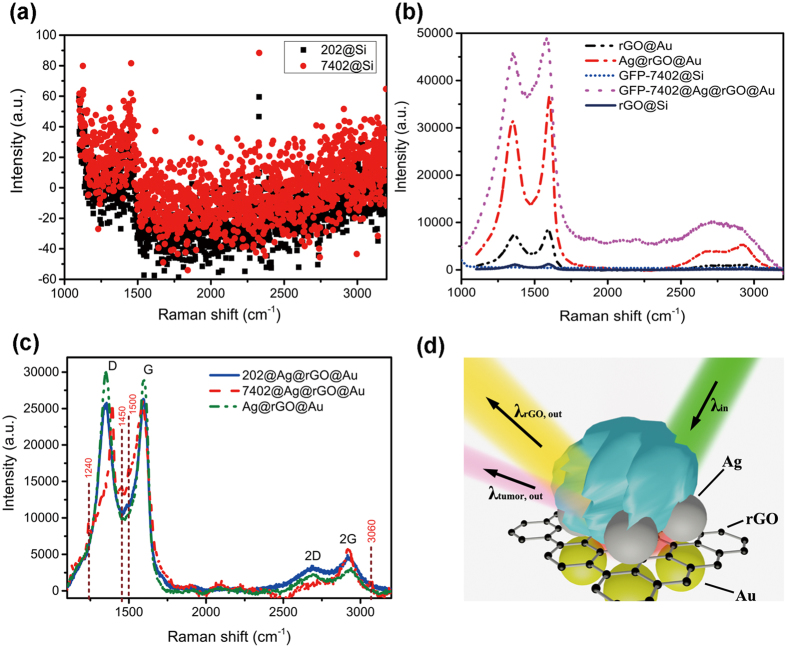
Identification and detection of tumor cells via SERS of Ag@rGO@Au. (**a**) Conventional Raman spectra of tumor cells (7402, red circle) and normal live cells (202, black square) on the silicon slide, which are indistinguishable in the vibration information. (**b**) Raman spectra of rGO on Si slide, rGO on Au nanostructures, GFP labeling tumor cells on Si slide, GFP labeling tumor cells on G-SERS substrate and G-SERS substrate. (**c**) Raman spectra of the same tumor cells (7402, red dash line), normal live cells (202, blue solid line) in (**a**) on our G-SERS substrate and G-SERS substrate (green dash dot line). (**d**) Schematic of mechanism for tag-free detection and identification of tumor cells on the substrate of Ag@rGO@Au.

RGO is a novel material developed for biotechnological applications due to its good bio-compatibility and chemical interaction with the target molecules^27^,^28^,^29^,^30^,^31^,^32^,^33^. In order to find out whether the Ag@rGO@Au based SERS can be suitable for sensitive detection of tumor cells, we select typical human tumor cells, hepatocarcinoma cells (BEL-7402) and human normal liver cells (202 hepatocytes). In general, these two kinds of cells are indistinguishable in Raman spectroscopy and hard to be identified as shown in Fig. 4a. Usually, the tumor cells are labeled by green fluorescent protein (GFP) during cell culture and are commonly observed under fluorescence microscope. We firstly locate the labeled tumor cells on our G-SERS substrate, as shown in Figs 4b and S5. The tumor cells on the Si slide don’t show any characteristic signals in the range of 1000–3200 cm^−1^ in the Raman spectra. Once the labeled tumor cells are placed onto the Ag@rGO@Au substrate, significant differences in Raman spectra can be observed surprisingly, which could be the direct evidence for tumor cells. In addition to the increases in intensities, widths and shapes of D and G peaks have also varied compared to corresponding peaks for Ag@rGO@Au. Especially for D peak, an obvious wavenumber shift of 25 cm^−1^ as well as spectral narrowing are observed. The distortion in characteristic peaks of graphene indicates the chemical interaction between rGO and tumor cells and affects the Femi level of graphene indicated in Raman spectra^34^. Moreover, additional satellite peaks around 1200 and 1500 cm^−1^ appear in the Raman spectra corresponding to the structural information of tumor cells shown in Fig. S5^35^,^36^. However, the photoluminescence of GFP, locating in range from 511 to 527 nm^37^,^38^, is also enhanced by hot spots raised by Au and Ag nanostructures, which covers a lot of structural information of cells for more sensitive detection. Since the process of label in GFP is complex and high-cost, it is thus interesting to explore the possibility of tag-free detection of tumor cells using this sandwich system of Ag@rGO@Au substrate exhibits coexistence of strong EM and CM enhancements. Herein, the tumor cells without GFP label and the normal cells as reference are located on the Ag@rGO@Au substrate in the photograph (inset of Fig. S6) and the conventional Raman spectra are obtained in Figs 4c and S6. The Raman spectrum of tumor cells on the Ag@rGO@Au (7402@Ag@rGO@Au) is dramatically different from both spectra of normal cells on the same substrate (202@Ag@rGO@Au) and the substrate (Ag@rGO@Au). The distortions of characteristic peaks (such as D, G, 2D and 2 G peaks) of graphene can be obtained even with 100 times lower concentrate and 10 times lower incident power as shown in in Fig. S6. Obvious satellite peaks around 1240, 1450, 1500 and 3060 cm^−1^ assigned to tumor cells can also be clearly observed in the sample of 7402@Ag@rGO@Au^3^,^18^,^35^,^36^. These results indicate that the Raman differences can be used to direct and fast identification for tag-free tumor cells. In this sense, the rGO could be regarded as roles of both substrate and bio-tags for detection and identification of tumor cells, providing the possibility to reduce the complex modification and cost for labeled tumor cells. Consequently, The G-SERS substrate of Ag@rGO@Au could be a good candidate for tag-free detection and identification of tumor cells simultaneously contributes to improvement of sensitivity and the discovery of the characteristic peaks of tumor cells by both of EM and CM.

In summary, we have designed and fabricated a G-SERS system based on Ag@rGO@Au sandwich structure, and an enhancement factor as high as 70 for rGO Raman signals has been obtained. The Ag@rGO@Au system has strong EM enhancement from coupling of LSPs by Ag and Au nanostructures, and GFP-labeled tumor cells could be detected on the Ag@rGO@Au based G-SERS substrate according to the enhancement and distortion of D and G peaks of graphene. Most importantly, the Ag@rGO@Au system also obtains strong CM enhancement from charge transfer between rGO and nearby target molecules. As a result, sensitive molecules information can be obtained, and tag-free tumor cells and normal cells could be distinguished clearly by Raman variation of characteristic peaks owing to the interaction with rGO which is located on the substrate of Ag@rGO@Au. In total, the G-SERS substrate of Ag@rGO@Au shows good potential as an efficient tool for rapid detection and identification of tag-free tumor cells.

## Methods

### Fabrication of Au@Si, rGO@Au and Ag@rGO@Au

Silicon wafers were firstly washed n deionized water, acetone, and isopropyl alcohol by sonication each for 15 min and then blow-dried by clear dry air (CDA). Au (99.999%, ZhongNuo Advanced Material (Beijing) Technology Co., Ltd) was deposited on the silicon wafers by E-beam evaporator (Syskey Co., Ltd) at 0.5 A s^−1^ in different time to obtain Au films with various detective thicknesses (1 nm, 3 nm, 5 nm and 10 nm). The as-prepared sample was named as Au@Si. The fabrication process of rGO film on Au@Si (named as rGO@Au) is as follows. Firstly, graphene oxide (GO) was prepared by a modified Hummers’ method as described in previous works^20^,^21^,^22^. Then the GO powder was suspended in deionized water by ultrasonication, resulting GO concentration of ~6.25 mg·mL^−1^. The substrates for graphene films were prepared using ~8 × 10 mm square Au@Si slides. Reproducible and uniform graphene films can be prepared by a spin-coating method. Typically, the substrate was completely covered with sufficient amount of the GO solution, and spin-coated at 500 rpm for 10 s followed by 2000 rpm for 30 s. The spin-coated GO films were heated in a quartz tube furnace under a continuous Ar flow as protective gas with a heating procedure as follows. Firstly the GO films were heated up to 300 °C at a rate of 5 °C·min^−1^, then to 950 °C at a rate of 20 °C·min^−1^ and maintained at 950 °C for 0.5 h, and finally cooled down to room temperature to obtain reduced graphene oxide (rGO) films. The as-prepared sample was named as rGO@Au. Ag (99.999%, ZhongNuo Advanced Material (Beijing) Technology Co., Ltd) nanoparticle film with a thickness of 3 nm was deposited on the rGO@Au wafers by E-beam evaporation at a certain rate of 1 A s^−1^. The as-prepared sample was named as Ag@rGO@Au.

### Raman spectrum, scan electron microscopy (SEM) and EDX mapping analysis

Raman spectrum was obtained on Renishaw micro-Raman spectroscopy system using visible excitation laser at 514.5 nm, 20 mW. The rGO on the surface was recognized according to the contrast to the substrate in the optical microscope. SEM images and EDX mapping of the samples were obtained on a field emission scanning electron microscope (S4700, Hitachi) with an accelerating voltage of 5 kV and eLINE Plus (Raith nanofabrication) with an accelerating voltage of 10 kV. By using different instruments to monitor the morphologies of Au nanostructures in different places, the Au nanostructures with detective thicknesses of 1 nm, 3 nm, 5 nm, and 10 nm, are almost in the same morphology correspondingly, indicating that the Au nanostructures could be fabricated uniformly in large area and it is conveniently suitable for application in large area.

### Atomic force microscopy (AFM) analysis

Samples for AFM analysis were prepared by spin-coating GO aqueous dispersion (0.25 mg mL^−1^) onto a freshly cleaved mica surface (500 rpm for 20 s followed by 2000 rpm for 30 s) and drying in air. Typical tapping-mode AFM measurements were conducted on Dimension Icon (Bruker Co. Ltd). The average size of the GO sheets is in the range of tens of μm and the height difference between the steps is ~1 nm, corresponding to a typical thickness of an individual GO sheet^22^.

### Reflection spectra

The spectra of samples were obtained in a typical home-made optical path. The reflectance was calculated based on the reference sample of Ag with thickness of 100 nm, deposited on Si via evaporation in electron-beam at the rate of 0.5 A s^−1^.

### Numerical calculation for localized electric field

Firstly, the SEM image in Fig. S2 was cut into a fixed size of 100 × 100 nm^2^ and promoted the contrast between the metal nanoparticles and Si substrate. Then, the prepared image was imported into commercial numeric calculation software (COMSOL Multiphysics). Optical properties of materials and air were defined according to the contrast, and perfect magnetic conductor and perfect electric conductor were used to describe the loss-free boundaries. Transition boundary condition was used to model the thickness of the nanostructure. Electric field distribution on the surface materials was calculated under incident of 514 nm. The near-field enhancement was defined according to average of |*E*/*E*_0_|^4^ under a distance of 10 nm between the detective plane and surface of imported image, in which *E*_0_ is the electric field in the non-structure and *E* is the electric field in the structure. We define the property of Ag at the wavelength of 514.5 nm by the permittivity of −100 + 2*i. In the calculation, the Drude model is employed to describe the optical property of Au in our previous work^25^. Because the Drude modal can’t perfect express the optical properties of gold when the size comes down to nanoscale, the model has its limitations in giving a comprehensive explanation of the measurement data. However, it will provide certain degree of insights into the experimental observation, showing that the localized field enhancement can be optimized by adjusting the gap between particles. The description of Drude model is in the following equation (1), where ω_p_ = 1.3699 × 10^16^ Hz, and r = 1.0644 × 10^13^ Hz^26^.


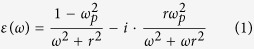


### Label-free detection and identification for tumor cells

The substrate used for detection and identification is Ag@rGO@Au with the Au thickness of 5 nm. Normal and tumor cells (202 hepatocytes and BEL-7402 hepatocarcinoma cells) are obtained from Laboratory Animal Center in Sun Yat-Sen University. One kind of tumor cell is labeled with green florescent protein (GFP) during cell culture which is firstly used for detection and the other label-free cells are investigated for identification. The process of experiment is detailed as follow. Firstly, cell culture fluid was removed and washed away once by pancreatic enzymes followed by deionized water for three times. Then, the cells were processed by pancreatic enzymes for cells desorption from sample bottles and resolved in deionized water (40 mL) to form cell solution. Syringe needle was used to move the cell solution to target substrate and dried by blower at low temperature.

## Additional Information

**How to cite this article**: Yi, N. *et al.* A hybrid system with highly enhanced graphene SERS for rapid and tag-free tumor cells detection. *Sci. Rep.* **6**, 25134; doi: 10.1038/srep25134 (2016).

## Supplementary Material

Supplementary Information

## References

[b1] Chen S., Li X., Zhao Y., Chang L. & Qi J. Graphene oxide shell-isolated Ag nanoparticles for surface-enhanced Raman scattering. Carbon 81, 767–772 (2015).

[b2] Li X., Choy W. C., Ren X., Zhang D. & Lu H. Highly intensified surface enhanced Raman scattering by using monolayer graphene as the nanospacer of metal film–metal nanoparticle coupling system. Adv. Funct. Mater. 24, 3114–3122 (2014).

[b3] Sha M. Y., Xu H., Natan M. J. & Cromer R. Surface-enhanced Raman scattering tags for rapid and homogeneous detection of circulating tumor cells in the presence of human whole blood. J. Am. Chem. Soc. 130, 17214–17215 (2008). 19053187 10.1021/ja804494m PMC2648857

[b4] Harmsen S. *et al.* Surface-enhanced resonance Raman scattering nanostars for high-precision cancer imaging. Sci. Transl. Med. 7, 271ra7 (2015). 10.1126/scitranslmed.3010633 PMC4414254 25609167

[b5] Xing F. *et al.* Ultrasensitive flow sensing of a single cell using graphene-based optical sensors. Nano Lett. 14, 3563–3569 (2014). 24793578 10.1021/nl5012036

[b6] Li J. F. *et al.* Shell-isolated nanoparticle-enhanced Raman spectroscopy. Nature 464, 392–395 (2010). 20237566 10.1038/nature08907

[b7] Chu Y., Banaee M. G. & Crozier K. B. Double-resonance plasmon substrates for surface-enhanced Raman scattering with enhancement at excitation and stokes frequencies. ACS Nano 4, 2804–2810 (2010). 20429521 10.1021/nn901826q

[b8] Ciracì C. *et al.* Probing the ultimate limits of plasmonic enhancement. Science 337, 1072–1074 (2012). 22936772 10.1126/science.1224823 PMC3649871

[b9] Marrucci L., Manzo C. & Paparo D. Optical spin-to-orbital angular momentum conversion in inhomogeneous anisotropic media. Phys. Rev. Lett. 96, 163905 (2006). 16712234 10.1103/PhysRevLett.96.163905

[b10] Xie L., Ling X., Fang Y., Zhang J. & Liu Z. Graphene as a substrate to suppress fluorescence in resonance Raman spectroscopy. J. Am. Chem. Soc. 131, 9890–9891 (2009). 19572745 10.1021/ja9037593

[b11] Bruna M. & Borini S. Optical constants of graphene layers in the visible range. Appl. Phys. Lett. 94, 031901 (2009).

[b12] Guinea F. & Horovitz B. & Doussal, PLe. Gauge fields, ripples and wrinkles in graphene layers. Solid State Commun. 149, 1140–1143 (2009).

[b13] Huang S. *et al.* Molecular selectivity of graphene-enhanced Raman scattering. Nano Lett. 15, 2892–2901 (2015). 25821897 10.1021/nl5045988

[b14] Ling X. *et al.* Can graphene be used as a substrate for Raman enhancement? Nano Lett. 10, 553–561 (2010). 20039694 10.1021/nl903414x

[b15] Li D. *et al.* Selective capture and quick detection of targeting cells with SERS-coding microsphere suspension chip. Small 11, 2200–2208 (2015). 25597293 10.1002/smll.201402531

[b16] Lu C. H., Yang H. H., Zhu C. L., Chen X. & Chen G. N. A graphene platform for sensing biomolecules. Angew. Chem. 121, 4879–4881 (2009). 10.1002/anie.200901479 19475600

[b17] Wang L., Yang F. H., Yang R. T. & Miller M. A. Effect of surface oxygen groups in carbons on hydrogen storage by spillover. Ind. Eng. Chem. Res. 48, 2920–2926 (2009).

[b18] Goncalves G. *et al.* Surface modification of graphene nanosheets with gold nanoparticles: the role of oxygen moieties at graphene surface on gold nucleation and growth. Chem. Mat. 21, 4796–4802 (2009).

[b19] Yu X. *et al.* Tuning chemical enhancement of SERS by controlling the chemical reduction of graphene oxide nanosheets. ACS Nano 5, 952–958 (2011). 21210657 10.1021/nn102291j

[b20] Marcano D. C. *et al.* Improved synthesis of graphene oxide. ACS Nano 4, 4806–4814 (2010). 20731455 10.1021/nn1006368

[b21] Zhang L. *et al.* Controlled synthesis of few-layered graphene sheets on a large scale using chemical exfoliation. Carbon 48, 2367–2371 (2010).

[b22] Wu Y. *et al.* Three-dimensionally bonded spongy graphene material with super compressive elasticity and near-zero Poisson’s ratio. Nat. Commun. 6, 6141 (2015). 25601131 10.1038/ncomms7141

[b23] Díez-Betriu X. *et al.* Raman spectroscopy for the study of reduction mechanisms and optimization of conductivity in graphene oxide thin films. J. Mater. Chem. C. 1, 6905–6912 (2013).

[b24] Ling X. *et al.* Raman enhancement effect on two-dimensional layered materials: graphene, h-BN and MoS_2_. Nano Lett. 14, 3033–3040 (2014). 24780008 10.1021/nl404610c

[b25] Yi N., Liu Z., Sun S., Song Q. & Xiao S. Mid-infrared tunable magnetic response in graphene-based diabolo nanoantennas. Carbon 94, 501–506 (2015).

[b26] Ordal M. A., Bell R. J., Alexander R., Long L. & Querry M. Optical properties of fourteen metals in the infrared and far infrared: Al, Co, Cu, Au, Fe, Pb, Mo, Ni, Pd, Pt, Ag, Ti, V, and W. Appl. Opt. 24, 4493–4499 (1985). 18224235 10.1364/ao.24.004493

[b27] Chang H. X., Tang L. H., Wang Y., Jiang J. H. & Li J. H. Graphene fluorescence resonance energy transfer aptasensor for the thrombin detection. Anal. Chem. 82, 2341–2346 (2010). 20180560 10.1021/ac9025384

[b28] Kravets V. G. *et al.* Singular phase nano-optics in plasmonic metamaterials for label-free single-molecule detection. Nat. Mater. 12, 304–309 (2013). 23314104 10.1038/nmat3537

[b29] Shao Y. Y. *et al.* Graphene based electrochemical sensors and biosensors: a review. Electroanal. 22, 1027–1036 (2010).

[b30] He S. J. *et al.* A graphene nanoprobe for rapid, sensitive, and multicolor fluorescent DNA analysis. Adv. Funct. Mater. 20, 453–459 (2010).

[b31] Zhou M., Zhai Y. M. & Dong S. J. Electrochemical sensing and biosensing platform based on chemically reduced graphene oxide. Anal. Chem. 81, 5603–5613 (2009). 19522529 10.1021/ac900136z

[b32] Mohanty N. & Berry V. Graphene-based single-bacterium resolution biodevice and DNA transistor: interfacing graphene derivatives with nanoscale and microscale biocomponents. Nano Lett. 8, 4469–4476 (2008). 19367973 10.1021/nl802412n

[b33] Koenig S. P., Wang L. D., Pellegrino J. & Bunch J. S. Selective molecular sieving through porous graphene. Nat. Nanotech. 7, 728–732 (2012). 10.1038/nnano.2012.162 23042491

[b34] Xu H., Xie L. M., Zhang H. L. & Zhang J. Effect of graphene fermi level on the Raman scattering intensity of molecules on graphene. ACS Nano 5, 5338–5344 (2011). 21678950 10.1021/nn103237x

[b35] Hawi S. R. *et al.* Characterization of normal and malignant human hepatocytes by Raman microspectroscopy. Cancer Lett. 110, 35–40 (1996). 9018078 10.1016/s0304-3835(96)04455-2

[b36] Qian X. *et al.* *In vivo* tumor targeting and spectroscopic detection with surface-enhanced Raman nanoparticle tags. Nat. Biotechnol. 26, 83–90 (2007). 18157119 10.1038/nbt1377

[b37] Tsien R. Y. The green fluorescent protein. Annu. Rev. Biochem 67, 509–544 (1998). 9759496 10.1146/annurev.biochem.67.1.509

[b38] Ormö M. *et al.* Crystal structure of the Aequorea victoria green fluorescent protein. Science 273, 1392–1395 (1996). 8703075 10.1126/science.273.5280.1392

