# Supplementary material for: A hybrid system with highly enhanced graphene SERS for rapid and tag-free tumor cells detection
Source: Sci Rep. 2016 Apr 27;6:25134. doi: 10.1038/srep25134 (PMC4846833; doi:10.1038/srep25134)
Supplement: Supplementary Information [file srep25134-s1.pdf]

Supporting Information for

A hybrid system with highly enhanced graphene SERS for rapid and tag-free tumor cells detection

Ningbo Yi[a], Chen Zhang[a], Qinghai Song[b,]*, Shumin Xiao[a,]*

[a] *Integrated Nanoscience Lab, Department of Material Science and Engineering, Shenzhen Graduate School, Harbin Institute of Technology, Shenzhen, China, 518055*

[b] *Integrated Nanoscience Lab, Department of Electronic and Information Engineering, Shenzhen Graduate School, Harbin Institute of Technology, Shenzhen, 518055, China*

*Corresponding author: S. X., shumin.xiao@hitsz.edu.cn, Q. S., Qinghai.song@hitsz.edu.cn

Fig. S1 to S6

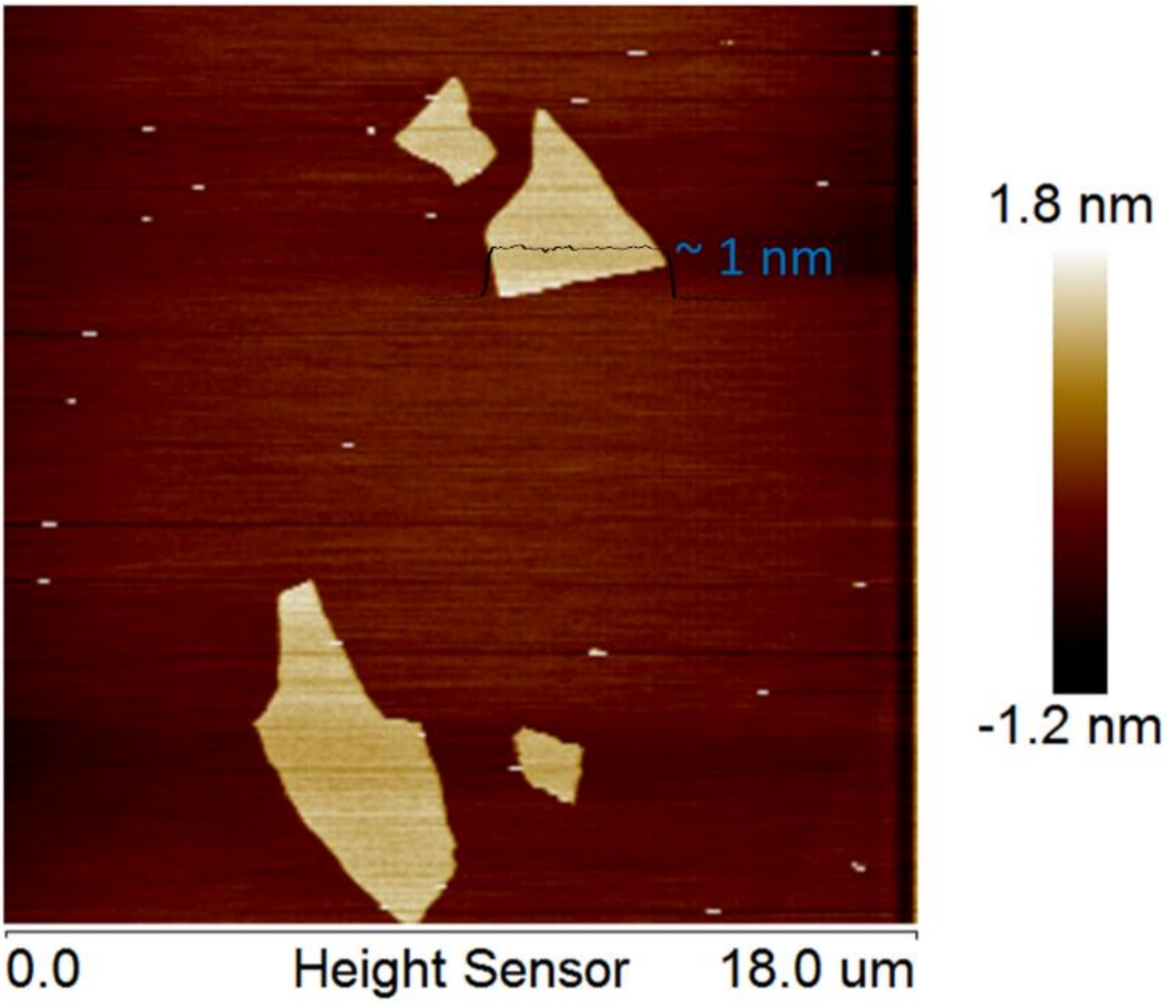

Fig. S1. A typical tapping-mode AFM image of GO sheets. The height difference between the steps is ~1 nm, corresponding to a typical thickness of an individual GO sheet.

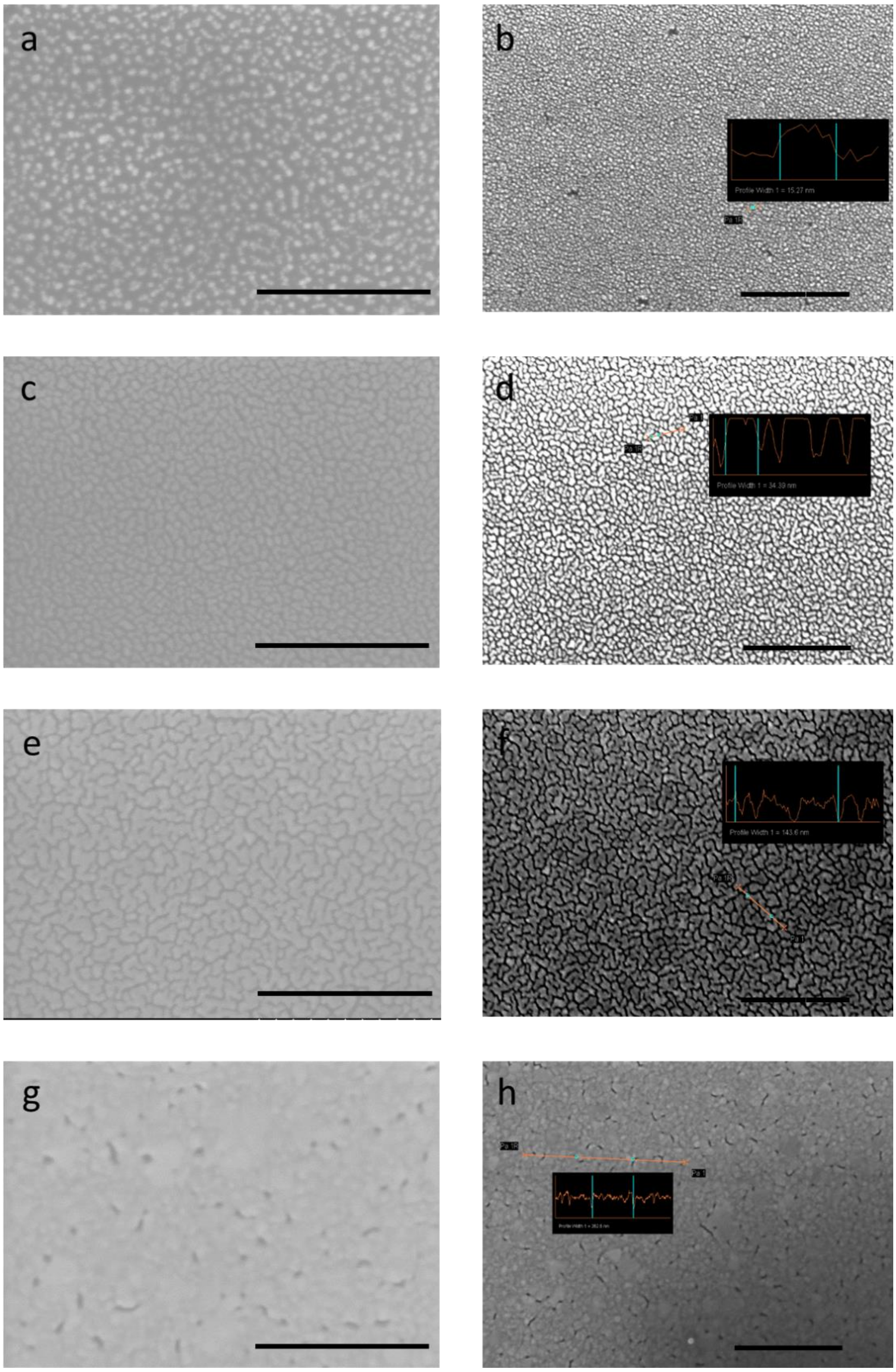

Fig. S2. SEM images of Au nanostructures with different detective thicknesses of 1 nm (a, b), 3 nm (c, d), 5 nm (e, f) and 10 nm (g, h). The domain size of Au nanostructures varies from ~15 nm to ~200 nm with increased detective thickness. The scale bar: 500 nm. The inset of b, d, f and h are marked as the domain sizes with different detective thicknesses, which change form tens nanometers to hundreds nanometers.

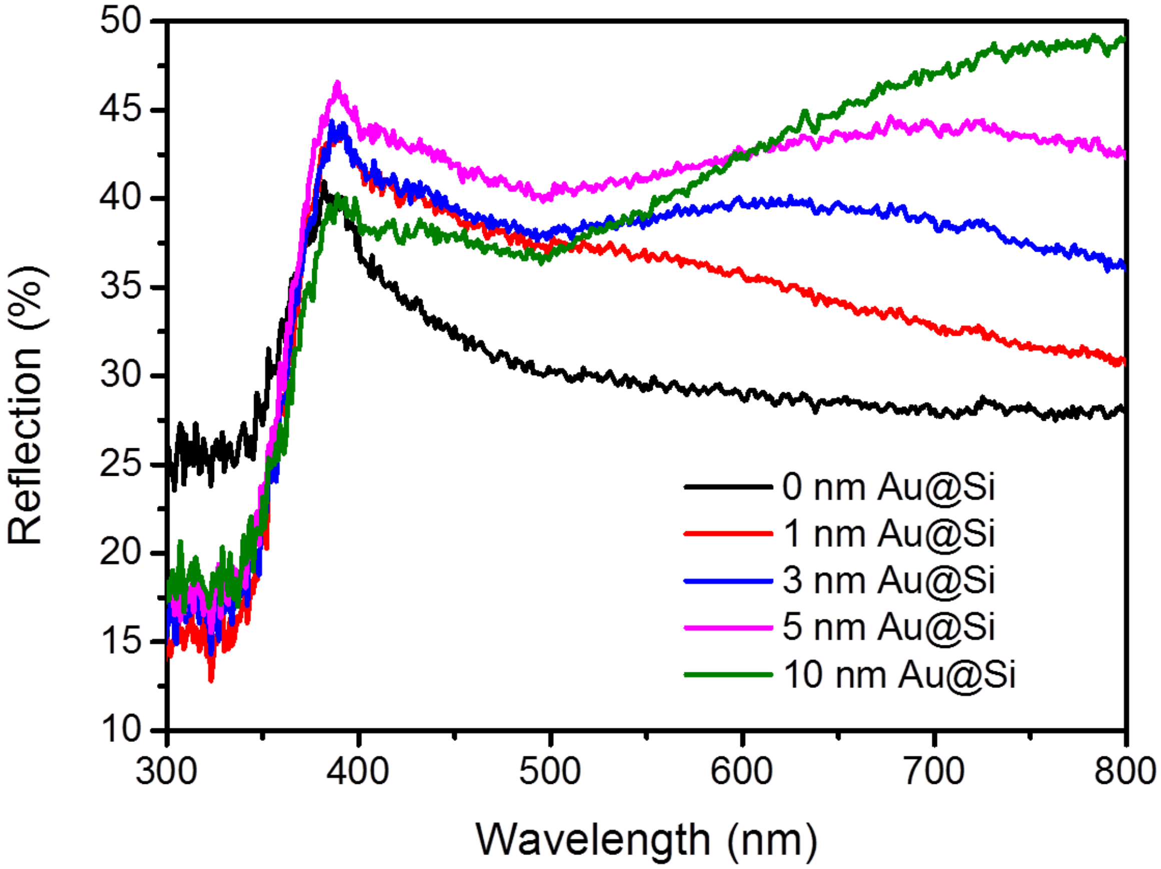

Fig. S3. The reflectance spectra of Au nanostructures in different detective thicknesses. The localized field is especially around the working wavelength of 514 nm.

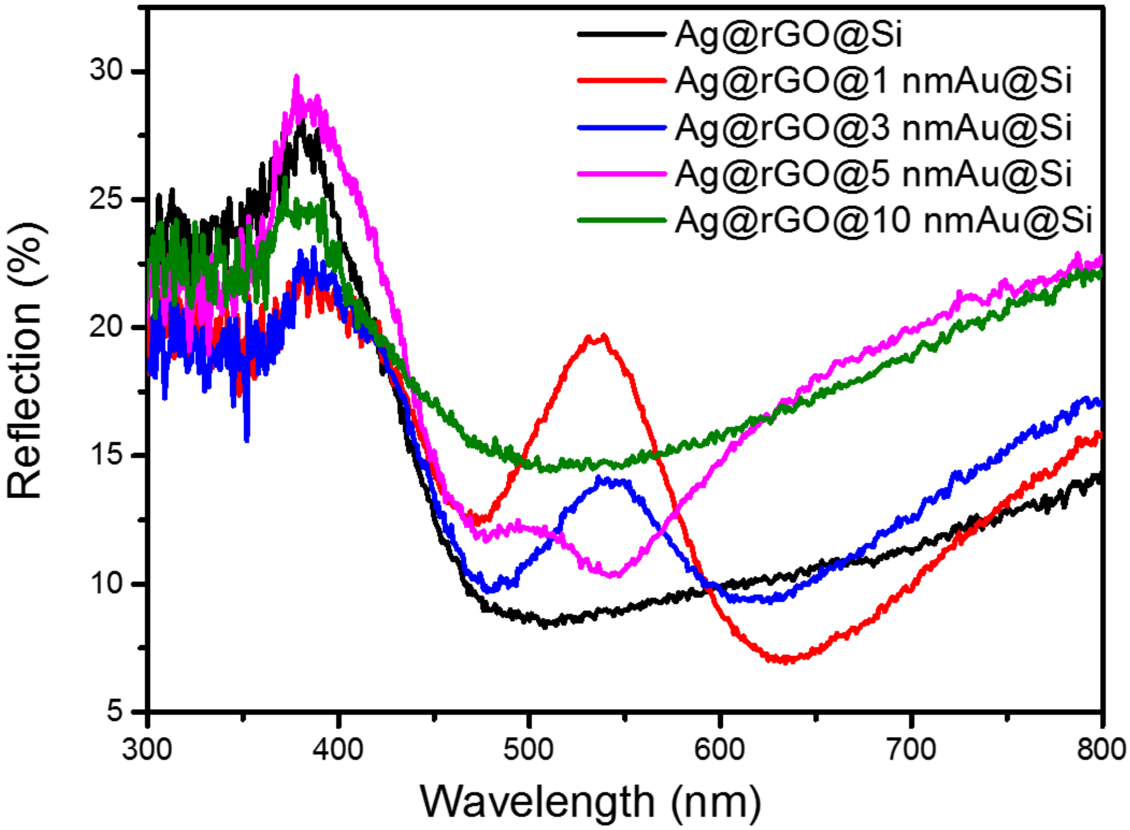

Fig. S4. Reflectance spectra of G-SERS substrates with different detective thicknesses of Au nanostructures. Introduce of Ag nanostructures can effectively enhance localized field and couples with Au nanostructures to form higher enhancement. Moreover, surface plasmonic forms to induce the adsorption peaks around target work wavelength of 514.5 nm.

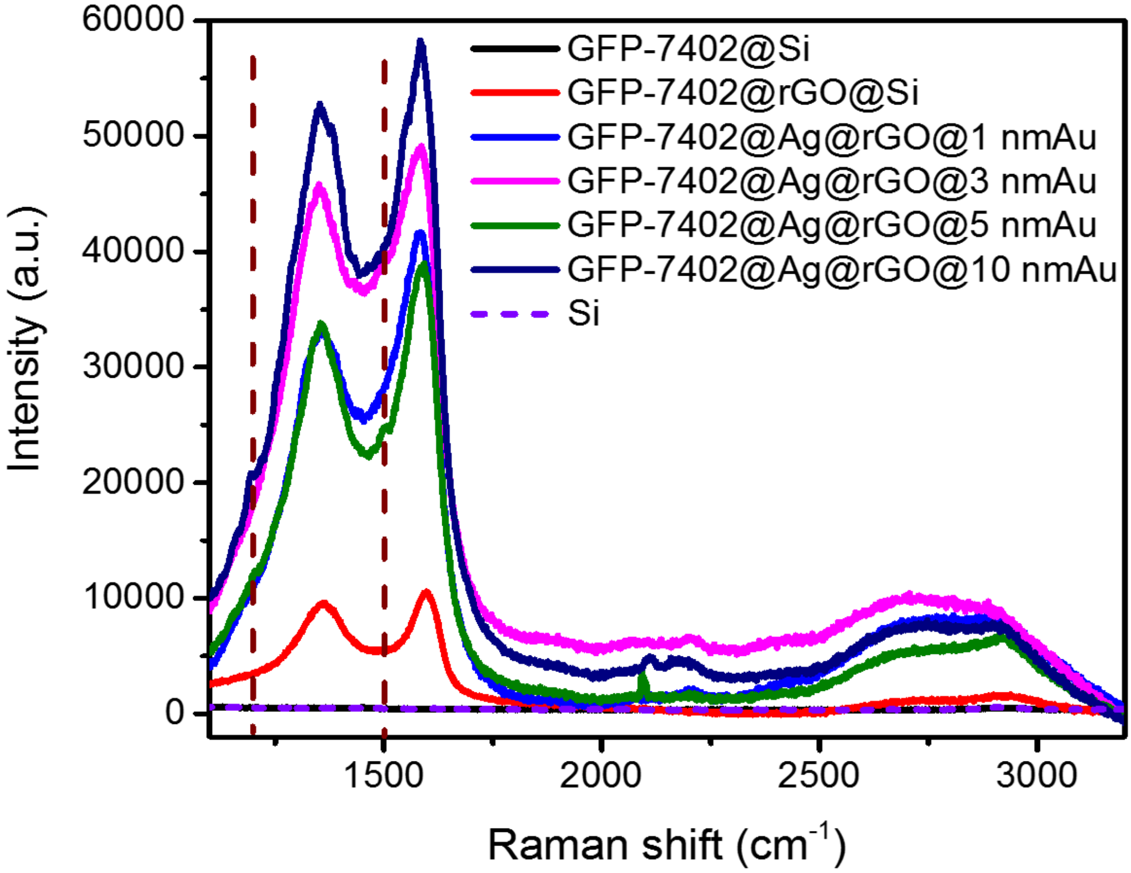

Fig. S5. Graphene enhanced Raman spectra (G-SERS) of tumor cells labeled by green fluorescent protein on the substrate of Ag@rGO@Au with different detective thicknesses of Au nanostructures. It is surprising that the D and G peaks get much higher enhancement, the peaks become wide and distortion of shape because of the chemical interaction between rGO and tumor cells in contrast to those of Ag@rGO@Au, which is the direct evidence for tumor cells.

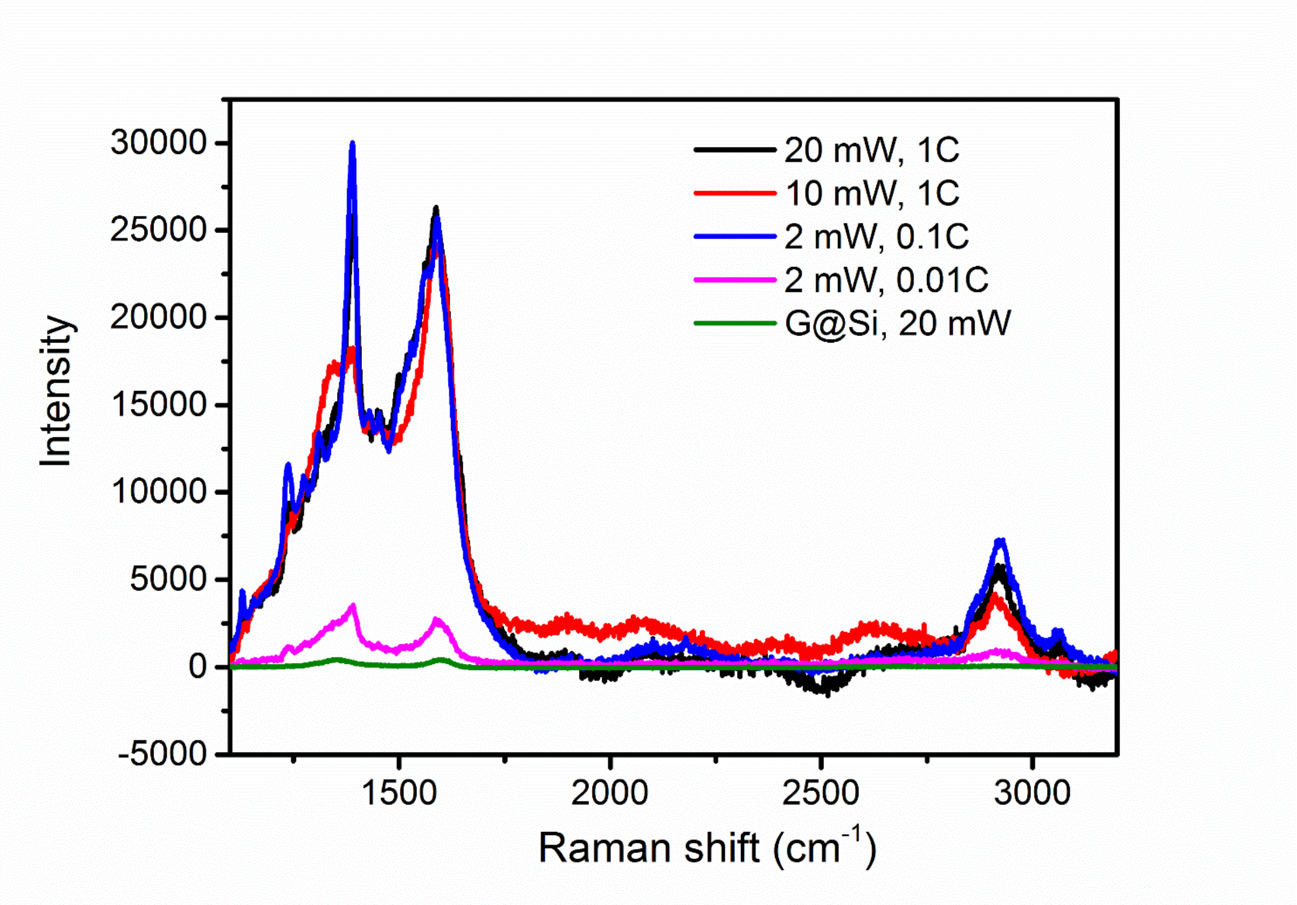

Fig. S6. G-SERS of label-free tumor cells on substrate of Ag@rGO@5 nm Au in different power of incident laser with the decreased concentration of tumor cell (normalized in the initial concentration). The introduce of tumor cells cause the discovery of satellite peaks existence around 1240, 1450, 1500 and 3060 cm^{-1} corresponding to tumor cells addictive and the characteristic peaks of graphene exist, which can be used to the direct identification for the normal cells and tumor cells. In addition, G-SERS substrate of Ag@rGO@Au in use is demonstrated to be a label-free tool to detect and identify the tumor cells up to a low concentration of cell culture in low incident power.
